# Academy of Stomatology

**Published:** 1905-09

**Authors:** 

**Affiliations:** Academy of Stomatology


### ACADEMY OF STOMATOLOGY.

AT the regular monthly meeting of the Philadelphia Academy of Stomatology, being the tenth anniversary of the founding of the society, a banquet was given at the University Club, Philadelphia, on the evening of April 25, 1905, at which many prominent members of the profession were guests. The President, Dr. I. N. Broomell, introduced Professor R. H. Hofheinz, of Rochester, N. Y., who read a paper entitled "Extension of Preliminary Education *versus* Extension of the Dental Course."

(For Professor Hofheinz's paper, see page 569.)

*Dr. R. H. Hofheinz.*—I rise with a feeling of profound sorrow for you all. If the Latin adage, "Plenum venter non studet libenter," which means that a full belly does not like to study, is true, I think it is also true that a man who enjoys a good dinner does not like to listen to dry discourses afterwards. It is almost an infliction, and if any one of you suffer from dyspeptic troubles to-morrow, I beg you to charge it to Drs. Kirk and Darby. It is the fault of Dr. Kirk that I came here, and the subject is the fault of Dr. Darby. I am almost afraid before you get through you will all feel like the cook who had been employed at the Oxford University. A number of the students complained to the dean, saying, "We cannot stand John's cooking any longer." The dean finally went to the cook and said, "John, the students are complaining about your cooking; they say it is very bad." "Never mind," replied the cook, "they tell me just the same thing about your lectures, Mr. Dean."

(The paper was then read.)

DISCUSSION.

*Dr. Broomell.*—We have listened to this very instructive paper on dental education and I take pleasure in calling on Dr. E. C. Kirk, who will open the discussion.

*Dr. E. C. Kirk.*—I am sure that I voice the general sentiment of the Academy when I say that we have listened with extreme interest to this thoughtful and suggestive paper by Professor Hofheinz. It is extremely instructive, and I feel that there is no question before the dental profession to-day so great in its possibilities and so pregnant with results to the future as the question of education. I was called upon at the last moment to open the discussion of this paper, and I have only had opportunity to glance hastily through it this afternoon, in the midst of distractions, so that I have hardly had time to digest all that has been brought before us; yet there are some points which have fastened themselves in my mind, and to them I would like to direct your attention.

I wish to say, in the first place, that I am in general agreement with the argument which the essayist has presented, but I wish to go perhaps a little farther. I want to call your attention to the difference in the attitude of human thought regarding what constitutes education as between our present period and that period to which he referred as the period of the middle ages I doubt, unless you have gone into the subject with some care, whether you have a clear conception of the state of mind or condition of human education at the period to which the essayist has referred in the sixteenth century and even later, and as an illustration which may serve to fasten in your thought the attitude of those who were considered educated at that period, I have brought with me to-night the original record of a controversy which flourished just at the close of the sixteenth century relative to a matter which comes very near to us as dental practitioners. In a book which I have here, which is a German translation of the original publication which appeared in London in 1595, we have the record of an investigation made by a learned professor of medicine and a teacher of his art, with reference to a phenomenon which occurred in Silesia, Bohemia; this phenomenon was the case of a boy of whom it was said he had erupted a golden tooth. To investigate this phenomenon the learned doctor made a pilgrimage to the town where the boy lived and personally investigated the boy's mouth. Having satisfied himself that there did exist in his alveolar border a tooth which was golden in its texture and appearance, and having satisfied himself by various tests that it was *de facto* a golden tooth, he wrote the account and learned treatise which I hold in my hand. In this treatise the author not only stated it to be a fact that the boy did have a golden tooth in his jaw, but he goes farther, and enters into a long and learned philosophical discourse to explain this wonderful natural phenomenon. It is not necessary for me to go over the details of it, but his conclusions were about as follows: The tooth was a supernatural thing in its origin; that it portended the return of the golden age; that because the tooth appeared at a time when certain astronomical conditions indicated or threw corroborative light on the case, it clearly portended that the whole world should be converted to the doctrines of Christianity, the return of the golden age and the ultimate overthrow or downfall of the Turkish Empire. I hold in my hand, again, a learned treatise written by a professor in a Scotch university, twenty years after the publication, in which he tears the whole fabric of the phenomenon to pieces, destroys the arguments of his predecessor, and shows that the father of this boy was nothing more than a mountebank, and that while there was a golden tooth, it was merely a shell crown or shell of gold placed over the tooth, and that there was nothing supernatural about it.

These two books are only two of a series of at least six or eight books that were published by learned men at that time dealing with that particular incident in that way. Think of it for a moment! That was only a little over three hundred years ago. It was a hundred years or more after the discovery of the American Continent by Columbus. In that period it was possible for men justly regarded as the learned men of their time to indulge in such fantastic speculations.

In the intermediate period a great change has come over the mind of humanity with reference to what constitutes education. Professor Hofheinz has clearly shown in his paper that the education of the middle ages was not education at all. It was an education that, I might say, was upon an inflated basis, inflated by ignorance and with little or nothing to give it a basis of truth. They concerned themselves with discovering what language the angels spoke, how old was Gabriel when he made the announcement to the virgin Mary, and other things which have been referred to. Now, we are questioning whether there are such things as angels, and developing an attitude of mind that is not satisfied unless it can be brought face to face with demonstration. That change has largely taken place since Herbert Spencer, fifty years ago, asked his epoch-making question, "What kind of education is of the most worth?" Education simply as an adornment is giving place to the education of usefulness which is derived from the study of observed phenomena.

In the past half-century since Spencer asked that question, we have seen the development of what has been called the scientific attitude of the mind toward the question of human knowledge.

Now, getting closer to the question involved in the title of the paper, and applying to it, as we may well do, Mr. Spencer's question, "What kind of knowledge is of the most worth?" we have to consider, as I understand it, the relative value for dental education of these two propositions, whether it is better to increase the preliminary requirements, or to increase the length of training in technical dentistry of the professional course. It is true, I believe, that improvement is necessary, is needful in the preparation of the mind for dentistry, and I would say that our whole system of preparatory education needs improving. The sentence quoted from President Butler, of Columbia University, of which I have here an abstract from the essayist's address, is as follows: "One of the best-known academies of the United States requires for admission only some knowledge of common school education, arithmetic, writing, spelling, and the elements of English grammar, and the average age of the pupils is sixteen and a half years. At that age the French and Germans have read Cicero and Socrates and Plato. It is very evident that at this point there is a tremendous waste in our educational system which must be remedied." Higher education is not to be discouraged, but we should concern ourselves with the question of this tremendous waste in our system of education and endeavor to stop it. I have, after a careful and thoughtful consideration of the matter, come more and more to believe that the "tremendous waste" is not so much in what children are instructed as it is in the manner of their instruction; it is not so much a question of how much they learn, but of how well they learn what they are taught. It is a question of method rather than of amount, or quality rather than quantity.

We are prone to say that education is the most important thing. I was interested in the reports of the recent yearly meeting of Friends in this city. Some of the questions propounded on this very problem of education they have been treating with that thoughtful consideration for which they are justly so well known, and it was asserted by some of their speakers that we are not sincere in this matter when we say that education is the most important thing. Do we, as a matter of fact, do the best, the utmost that we can do for the education of the young? Is it true that those who are devoting their lives to education are selected with reference to their ability, and are they given sufficient compensation to attract to the profession of teachers those who are qualified to teach, to educate? These are very important things. If we look over the records of the municipal system of education and compare it even with private schools, we find that the amount of compensation for teaching is meagre, and it does not bear favorable comparison with what is paid ordinary clerks. Many teachers who are engaged in teaching the young have less compensation than you pay to the cooks in your kitchen, and with that as the case you cannot attract those most capable of carrying out this work. On the other hand, we have the opportunity to judge of the system of preparatory education by the character of the product that comes to us as the material upon which we are to begin our work as educators for the dental profession. Some careful observation of that matter, some few years of experience in the study of the product which the high schools are turning into the dental colleges has convinced me that the system of education even in our high schools is also imperfect. There are indications there also of a waste of time in the matter of method and quality of instruction. The high school graduate is old enough to reason and to think precisely within the limits of his knowledge, but he comes to us as a confirmed memorizer, not as a disciplined thinker.

Let us bear in mind that education is not what a man knows, but it is the reflex of that knowledge expressed as character and the ability to use the increased power which education brings, which is the real educational result. I know in my own experience that many men who come to us even after high school graduation are wofully deficient in many points, and especially in knowledge of their mother tongue. Not a small proportion of the men who come to us from the high school to-day are ignorant of the elements of the English language. They have not that clear mental conception of the use of words which enables them to use words intelligently or to comprehend the meaning of words when they hear them spoken or read them. Therefore, I believe that the English teaching of our preparatory schools is deficient.

I would call attention, further, to a point which the essayist has brought out very markedly in his paper,—that the question of manual training is one which should be part of the preparatory training not only of the student who goes into dentistry, but should be a part of the education for students of the preparatory school in all departments. We have only begun to conceive the possibilities of manual training and its value; we speak of it sympathetically because we realize that it brings increased skill; but it means much more than that; it is a system of mind training, it is a system of nature study, a system of impressing on the perceptive faculties concrete things and not ideas about things. It is a system of education along the lines of least resistance. Therefore I would emphasize the necessity of manual training, not only for its value in producing finger-skill, but especially as a method of mind training. Dr. Hofheinz spoke approvingly of the advance step, as he calls it, which is to be taken by the regents of the city of New York with reference to increasing the number of academic counts required by law before a man in the State of New York may be permitted to enter a dental college, and that a four years' high school course will be required instead of a three years' course, and yet to my mind he neutralizes almost in the next sentence the value of the step by stating that these forty-eight counts may, in the future, be made up by selection from no less than seventy-six different subjects.

It seems to me the thing to be considered is not the quantity of things that a man may know, but how well he knows a certain number of selected things. I think the utilitarian idea in education is an essential point to be considered, and the training of the prospective students in lines which will subsequently be of use to them in taking up the dental course. I do not wish to be considered as being in a hypercritical attitude toward this question. I am in a critical attitude, but I do not wish to be regarded as an iconoclast,—of tearing down an existing system without offering a suggestion for its betterment. Therefore, in prescribing a course of instruction for a prospective dental student, I would first have him trained in his own language, and that means that he shall not only know English, but he shall also know some other language. I am a believer in the principle which was laid down by Goethe, referring to the study of languages, when he said,—

> "Wer keine fremde Sprache spricht,
> Kennt seine mutter Sprache nicht,"

which means that he who does not know a foreign language does not know his own. He should at least know one foreign language. I would have him know history, that is to say, the general history of the world's development. He must know something of history from its sociological stand-point. He must know mathematics, and why? He must have it for the development of the faculty of precise reasoning and the proper perception of magnitudes. He must learn it to be a fact that two and two make four, and not three and a half or five,—make four and nothing else. He must know science, at least a smattering; he should know enough of it so that he shall be able to comprehend the great fundamental generalizations of science. I plead for a science training so that he may get rid of the last fragment of superstition or mysticism about his work, and because from the training in science he shall be trained to observe phenomena and to assay them at their true value, and not on the inflated basis of ignorance or superstition or mysticism. If he has English, a training in mathematics, a knowledge of history, and a grounding in science, I will trust him to develop well under the dental course.

I have not yet come to the point of the essay. As I comprehend it, the essayist puts before us the problem as to whether it is best, under the circumstances, to lengthen the dental course and hold the preparatory education as at present, or to first increase preparatory education and hold the technical training at its present point. I believe in both of them. I think we need both things, and we need them very badly; but what I have said with reference to the difference between the quality of the preparatory education and its quantity I think will explain my position on that point. I believe it is quite possible, if our preparatory systems of education were so intelligently reorganized as to complete the course of training in three years of high school work, it would be sufficient to fully prepare men for studying dentistry; but to change the existing system of preparatory education is a task to which the cleaning out of the Augean stables would be a mere bagatelle. Possibly we shall have to live another generation before the necessity of changing the present system of preparatory education is realized so that efficiently trained students may be turned out from our schools in a minimum period of time.

I plead for the increased length of the dental course. The argument which has been quoted from the address of Dr. Brophy, that four years of six or seven months is less than a three years' course of nine months, is a specious argument. It was held up by those who advocated the establishment of a four years' course, that if we had four terms of any number of months, we would by that act have fastened the course down to four separate years, and then those institutions which desired to do so would have been able to maintain their four terms of nine months each, and there was nothing to prevent them from doing it. It is true that comparison of four years of six months to three years of nine months is to the disadvantage of the four years' course, but with four annual sessions those who were in favor of the four years' course would have been able to maintain satisfactorily the four annual sessions of nine months each.

I think we need the four years' course of instruction. The total amount of knowledge, the accumulation of data with reference to dentistry, that which constitutes dental knowledge to-day, cannot be put into the head of a dental student so that he can retain and utilize it in less time than four years. We are doing the best we can under the circumstances, but every one of us knows that we could do better with a four years' course of training. I have been told that the late Samuel D. Gross, years ago, when president of the Board of Trustees of the Pennsylvania College of Dental Surgery, was at one time in the institution waiting for a meeting of the trustees to convene, when, incidentally, a plumber's assistant came into the room with a kit of tools. Dr. Gross said to him, "Young man, how long does it take you to become a plumber?" He said, "Four years' apprenticeship." "Why," he said, "come around to the Jefferson Medical College, and we will make a doctor of you in two."

Is it not a wonderful condition of affairs that it takes less time to make a dental practitioner, who, if he is a conscientious man, must know the structure of the human body, its composition and its functions, thoroughly and well, and must erect upon that foundation a special technical training necessary for him to apply the science of dentistry in his profession, to know all that and to make a professional man in less time than it takes a plumber to become proficient. Are we acting sincerely in this matter? I think not. "A stream cannot rise higher than its source" has been said very truly. The source of this question of education is the attitude of the dental profession with regard to it, and just so long as the dental profession at large is willing to stand idly by and see men unfinished in their education turned into the ranks of dentistry, just so long will inferior education be the standard. When they are ready to take that up by enacting a standard that shall be in the form of a statutory law forbidding that these things shall be done, then we shall have a high standard in dentistry.

*Dr. Wilbur F. Litch.*—I wish first to thank the society for the kind and courteous invitation to be present this evening at this its tenth anniversary, thus giving me an opportunity to enjoy a most pleasurable evening and to listen to the excellent paper of Professor Hofheinz.

My individual society activities have been associated with another society of this city, one which has the distinction of being the oldest dental society in the world, and as a member of that organization I desire to extend to the Academy of Stomatology the felicitations of the old Pennsylvania Dental Association, the mother society, upon this auspicious occasion, and upon the success, prosperity, and usefulness of the Academy of Stomatology.

I have listened with great pleasure to Professor Hofheinz's paper, and the pleasure was the greater because the paper was not a controversial one in any sense, and because it was, as we might well have expected from its source, distinguished by clarity and breadth of vision. Its author has gone into the fundamental principles of education, and has shown us very clearly in what we are lacking in this country. As a teacher for more than a quarter of a century, it has been my fortune to have under me as dental students a large number of gentlemen from the other side of the water, especially from Germany, and I can testify, as can every other instructor, to the general superiority of their educational preparation over that which characterizes the average American student. The magnificent public school system of Germany is at once an honor to that country and somewhat a reproach to our own. It is the crowning glory of Germany that she has a school system so broad in its conception and operation, and so fruitful in results. German students come to us with trained minds, and, notwithstanding the difficulties of listening to technical instruction in a foreign tongue, grapple successfully with the subjects put before them. Not that they are ignorant of our own language by any means, but when they come here they are necessarily without that degree of skill which can only be acquired by considerable training of the ear.

We, in America, have advanced very markedly to-day beyond what was the standard a few years ago, when, as Dr. Kirk has remarked, a plumber's assistant could be taken into a medical school and graduated in two years, but much still remains to be desired. The enforcement of a uniform advanced educational standard for all sections of the country is attended with almost insuperable difficulties. This is a very large country. It contains many classes of people with varying social customs, standards, and possibilities. We have in the southern section of our country States which are just emerging from the devastation and wreckage brought about by civil war, States in which the educational system was very meagre before that conflict, reaching only a small percentage of the white population and, of course, being almost entirely withheld from the negro. These States are to-day doing heroic work in building up their educational system; but it can only be after long years of effort that education can be made as general and as advanced as in the Northern and Western States.

Dr. Hofheinz, in his address, spoke of high schools as having in a majority of instances not more than a three years' course. Not very long ago I obtained from official sources a list of high schools in the State of Pennsylvania having a four years' course, and to my very considerable astonishment the list embraced nearly two hundred schools. I had no idea that Pennsylvania was so rich in high schools. My enthusiasm was dampened, however, when I learned from the same official source that "except in a very few cities, the high schools are very small, with one, two, or three teachers, with courses of study not much, if any, beyond the grammar school course in Philadelphia." The explanation of this anomaly is that the amount of appropriation obtainable from the school fund of the State is dependent on the number of courses given, so the courses are multiplied, although the curriculum is not correspondingly extended. As is well known in Pennsylvania, we are, as compared with many other States of the Union, very backward. It has been officially stated that Pennsylvania stands twenty-third in the list of States as regards high school facilities and money expended for high school instruction.

There is, however, a determination to better this condition of things. As you are aware, the State Legislature has just passed a law by which the public education of the city of Philadelphia is largely taken out of the hands of politicians and placed in the hands of men who will devote their time and attention to education entirely free from political influences. The appropriation for schools has been fixed at a definite sum, and we can hope for a rapid improvement in educational affairs both in the city of Philadelphia and State of Pennsylvania.

These facts, of course, have a very important bearing upon the question of raising the standard of preliminary education as a requirement for admission to the dental schools. Desirable as it would be, I regard it as practically impossible, for the reasons I have stated, to enforce at the present time an entrance requirement, uniform for all the States, much higher than the present standard, a completed two years' high school course. Dental and medical schools are becoming more and more local in their attendance, and cannot successfully keep much in advance of the educational standards and possibilities of their constituency. Nevertheless, I am heartily in sympathy with the view of Professor Hofheinz that the elevation of that standard is the matter of primary importance in dental education. As Dr. Kirk has stated, many of our students holding high school certificates or diplomas come to us sadly deficient in power of analysis and in knowledge of the value of words and the correct use of the mother tongue. The fault is not with the dental college, but with the preparatory school. Colleges must accept students as they come to them and receive educational certificates at their face value. That manual training is not more general is another defect in our common school system. A professor in one of our Philadelphia manual training high schools recently remarked to me that he thought such schools a mistake; that manual training should be a part of every high school course and not restricted to special schools. The training of eye and hand in constructive work he regarded as a valuable educational asset for any boy. He thought, moreover, that the institution of special schools for manual training had cast a sort of stigma upon their students, the students pursuing the academic high school course regarding them as distinctly lower in the scholastic and even the social scale than themselves.

When we come to consider the real objects of education, we can hardly improve upon the analysis of Lord Bacon, that "studies are for delight, for ornament, and for use."

The student who engages in hard study simply for the delight it affords him is, I think, in modern life, very rare. Acquiring culture for culture's sake is not a pursuit which now appeals to the many. There is still a large contingent who take up studies simply as an adornment, to be able to say they know this or that language or that they are graduates of this or that school; but the fundamental thought of modern education is largely the utilitarian one. The high school diploma or degree in art or science is not only becoming a matter of social adornment, but the necessary equipment for a man who would succeed in life; and young men are more and more fully realizing that the great prizes of life even in commercial pursuits are going to the high school or technical school or college men. When this fact is once fully understood, we will have begun to build up upon utilitarian lines an educational system worthy the name. That time is rapidly aproaching, if not already here, and I look forward to the next few years as a period to be marked by the most rapid development in educational interests throughout the country, and have no doubt that as a reflection of that we shall be able in a few years not only to greatly advance the preliminary educational requirements of dental students, but if demanded by advances in dental art and science to still further extend the course of college instruction.

At the present time, I do not see the necessity for giving our students more physiology or chemistry or anatomy or materia medica than they are already receiving. The general consensus of opinion of those who advocate the four years' course is that it is required not so much for advanced theoretical as for further technical instruction, for another year of training in the manipulative process of operative and prosthetic work. If that, under existing conditions, were a practical thing, if it were possible to take the third-year student and pass him on to the fourth year, giving him a little further drilling in theory, but for the greater part of each day keeping him busy in practical work at the chair, then, I would say, by all means, give him the four years' course; it would be money in his pocket, for he would go out better prepared for his life work and more assured of success.

The difficulty in the city of Philadelphia, where we have a number of dental colleges and any number of "dental parlors," is to obtain patients, and it would be practically impossible to keep the fourth-year students busy at the chair each day and still do justice to the second- and third-year men.

We might, by display advertising and other dental parlor methods, make our clinics equal to the further demand, but that would be derogatory to the dignity of the school, a violation of professional ethics, and an injury and injustice to the dental profession of Philadelphia.

Conditions in the future may change, but at the present time that is my great objection to the four years' course.

*Dr. Kirk.*—Dr. Litch referred to the desirability of the advanced preliminary requirements. Before he relinquish the subject I should like to have him explain what his idea is regarding advanced preliminary requirements, whether the kind of subjects or the quality of training?

*Dr. Litch.*—My opinion is that they ought to be subjects, if not strictly germane to dentistry, at least in some degree aids to its study and practice as an art and science.

*Dr. Inglis.*—Dr. Hofheinz has opened the question whether preliminary education should be increased or whether the course should be increased, and in his paper he referred metaphorically to the education of boys. He would offer to two boys two cakes; to the lawyer he would offer one supposed to contain pabulum which would fit him to become a lawyer, and to the other, destined to a commercial life, he would offer a cake containing commercial pabulum. Now, boys in knickerbockers, or even older boys, often have no idea of pursuing the profession of dentistry unless their fathers happen to be dentists, and in the event of a sudden determination to enter upon it they must depend upon their general training.

With reference to early training with a special object in view, I am reminded of a question asked of Dr. Oliver Wendell Holmes as to how early a boy's education should begin. He replied that it should begin about a hundred years before the boy was born. I suppose he meant by that that his forebears, after a thorough education, would transmit a certain quality of mind which would enable him to take in pabulum which would probably fit him for his future work. Now, that a good preliminary education is of great value to a man in whatever walk of life he may take up I think will be granted by any one here. The fairest flowers undoubtedly come from cultivated fields, but the question arises whether the men and women entering the dental profession come with sufficient cultivation for us to take them only from those cultivated fields. We have a certain course for these men; we invite them to come and take the course with us, and we are compelled at present to accept within limitations such material as these persons bring to us. That, of course, brings up the question presented by Dr. Kirk as to the quality of their preliminary education. We are to decide as to whether those presenting have sufficient knowledge to enter the dental college. It is within my own experience at our own school that men have presented themselves who had not quite sufficient preliminary education to enter the college, and it was suggested by Dr. Singer that these men should take the general course, but make up certain subjects which were material; in other words, they entered with conditions.

Some of these men have become exceedingly good students, who turned out to be good mechanics, capable of acquiring the so-called theoretical branches. Now, if the standard of preliminary education had been rigidly enforced, they would have been rejected at the entrance examination. Of course, grossly incapable men should be prevented from entering the profession, but it is a question whether many apparently insufficiently educated have not sufficient intelligence to be received, with great hope of eventual apprehension of their subjects, and I wish to emphasize that point.

I think the New York State requirement of a sufficient number of counts earned upon a variety of subjects is a fair test of intelligence. After all, it is the only important thing, the ability of a man to apply his knowledge, which makes him proficient.

With respect to knowledge of foreign languages, I had occasion not long ago to raise a question in my own mind as to the word "post-extraction." We have had the word "post-mortem" for a long time, and I asked several college-bred men, one of them a Yale graduate, how to best construct the word. I also asked a gentleman who teaches Latin, and I got from him a dictum that "post-extraction" would be a very bad word, that it would be a combination of an English word with a Latin prefix. I asked another, and he had a different idea, and so it went on.

Bearing upon this I recall an incident which happened in my own family. At the time I was in South America acquiring some knowledge of the Portuguese. I sent for my family to come down, and on the way out my wife met a gentleman who was highly educated and who spoke six or seven languages. She told him I had advised her to study as much Portuguese grammar on the way out as she could. He said, "I would not do that; I would study French first. A knowledge of French will help you a great deal in your Portuguese." Now, then, we will suppose the word "star" in French and Portuguese. I question very much if the knowledge of the French word "etoile," or the Latin word "stella," would be of any great help in acquiring the Portuguese word "estrella."

In studying for my examination in Portuguese before the Dental board of Rio de Janeiro, I found that my familiarity with Latin names was of much value, but they were acquired from a study of them at the dental college, and not because I possessed any great memory of my Latin studies at school.

It seems to me that at present the best thing for the prospective dental student to do is to get as much education as he can and then go to college. I firmly believe that while a man should have a fair degree of education, it matters not so much what he has acquired, provided he is able to take up the study of dentistry intelligently, and the course should be so arranged that after he is once in, it will be difficult for him to get out, so that he may be as well educated in dentistry as possible.

As to whether the course should be lengthened depends, I think, on the man. It seems to me a three years' course is quite long enough for some men and not enough for others, and if that course can be arranged so that it will give a student the essentials of dental education, and if he is capable of acquiring these essentials in a course of three years, he should be permitted to go before the State Board, and if he is not prepared for that he should be held back until sufficiently proficient.

It seems to me that there is another important matter to be thought of, and that is the charges of the schools for dental education. I think college fees for education in dentistry are entirely too low when compared with other private school instruction. If the fees were raised to one hundred and fifty or one hundred and seventy-five dollars it would do more toward elevating the standard of education in our dental colleges than any other one thing.

*Dr. James Truman.*—If I understand the drift of Professor Hofheinz's paper, it is that it is essential to have a higher preliminary education. My experience has been that the higher the preliminary education is, the less satisfactory will be the dentist. Last year, in preparing a paper on this subject for the Dental Congress, I took the trouble, at the University of Pennsylvania, to go through the lists of those who had taken the practical course in the first year of the high school, the second year of the high school, and finally the diploma of the high school, and I found, by taking the average examination in the practical branches, that the men who had only the first year of the high school were as good as the men who went through the four years and received the high school diploma. The higher you go in preliminary training will there be less ability at the end of the fingers to accomplish practical work.

A great deal has been said here regarding the German schools. No one has a higher estimation of the German method of teaching than myself, because I was brought into close contact with it during a residence in Germany, and I am prepared to assert that in my opinion it is the best educational system that the world has ever produced, but the men that come over here from Germany to our schools with this highest education are defective, as a rule, in technical ability, and the reason is that they are too old when they begin to study dentistry. It is not their fault. The preliminary education has not helped them one particle in their practical work.

Allusion has been made by Dr. Hofheinz to the colleges of fifty years ago, and of the course then of four months. We had five months at that time, but all the students, so far as I remember them, had from three to five years with preceptors in dental offices before they matriculated, and then had superadded to that two years of college training, and those men, as a rule, have made a record in their profession quite equal to those with modern training.

I am not in opposition to preliminary education, but, on the other hand, am a strong advocate for the best possible, but I do not want it at the expense of practical ability.

I was surprised when the assertion was made that if we had four years, and the last year entirely devoted to practical work, that it would be absolutely impossible to give a hundred men practical instruction. We are doing that day after day in the University of Pennsylvania, with a hundred chairs filled all the time, and we could carry that on for the next five years if it were necessary to do it. There is something wrong somewhere.

This is a profound subject and I would like to talk on it more than five minutes, but I know very well the hour will not admit of it. We want the four years. The idea has been suggested that it is possible to secure as much from three years of a certain number of months as you can out of four years, but I believe that to be a fallacy. With the mass of study to be digested, it is utterly impossible to accomplish it in three years. The practical must be sacrificed in order to carry the student through the term. The men who brought about this change from four to three years are responsible for lowering dental education in this country.

*Dr. Robert H. Nones.*—Dr. Kirk's mention of Professor Gross's statement to the plumber that he would graduate him in medicine in a couple of years would have been more applicable had Dr. Gross told that plumber he could be taught dentistry in a couple of years, for the reason that the plumber had practical experience, and that is what counts.

Dr. Truman touched the point which occurred to me,—that is, the immense change which has taken place in the teaching of dentistry, even in my time. But a few years ago men took up the preparatory course with a preceptor, and Dr. Truman spoke of those men becoming better dentists than those of to-day. Why? Because very many of the men who were never fitted in any possible way to continue in dentistry and go to college were dropped before they reached it. Their preceptors very kindly told them to take up something else. To-day the colleges get many men who would not have entered the profession under the earlier method.

Then there is another point that has not been touched upon, and you have probably felt a little hesitancy in doing so, but, to use a very common expression, "colleges are not run to save souls." They are conducted, at least, to meet their expenses and to teach dentistry to the best of their ability. If the dental colleges were so endowed that they could select just what students they wished, what a grand thing it would be for the teachers, the profession, and for the public as well, but unfortunately such is not the case.

If I lived in New York State, I could most heartily agree with Professor Hofheinz in his belief, because the law compels us to do so many things we would not otherwise do. The law of New York says that you must do this, but the law of Pennsylvania and that of other States does not do so. For that reason, possibly, I cannot entirely agree with him. If I were not associated with college work at all, I might also agree with some of the expressions frequently made about colleges.

Dr. Inglis touched on a point relative to "students applying to the different colleges who should not be admitted without the proper preliminary credentials." I would like to ask if we have a right to turn away those who desire to study dentistry because they have not all the requirements? It becomes a serious point and a very vital one. Have we the right to refuse to teach a man dentistry because he has not at that time these preliminary requirements? I think that any one will admit that good environment, as a rule, tends to make better men. I have seen two dental students, one not having these preliminary requirements, —and have been surprised to see how that man has advanced, while the other, who possessed them, proved worthless. Dr. Truman also said that the "best-educated men make the poorest dentists." He does not mean any one to understand that, all other things being equal, the educated man would not turn out as good. I think, under the conditions stated, the man with the better education would certainly turn out the better dentist, but in ninety-nine cases out of a hundred, where he possesses the higher education, he has, in nearly every instance, sacrificed manual dexterity. Does dentistry rest entirely with the brain, or is most of it accomplished with the hands? Which of the two must be sacrificed, if either? These are points we naturally should consider.

I did not for a moment expect to say anything upon the subject, but am somewhat compelled to answer the charge of lowering the four years' course. I do not feel responsible for the three years' course, but we felt that a four years' course was not necessary. In advocating a return to the three years' course we could not have been absolutely in error, inasmuch as institutions which we all respect accepted the shorter course.

We had no intention of lowering the standard of dental education. If there is a school that is endeavoring and has endeavored in the past to advance dental education, to uphold the laws, and make better laws in the State of Pennsylvania, it is the institution with which I am connected. Whenever a four, five, or six years' course seems to be practical, there will be no college more willing to adopt it than ours.

We can make of some applicants dentists in a year, and others cannot be educated in fifty years. Some can be made with a grammar school education, and others not with a collegiate education.

The President called on Dr. Hofheinz to close the discussion.

*Dr. Hofheinz.*—It is too late to detain you any longer. I agree entirely with Dr. Truman that too much education will make a poorer dentist. I dwelt on over-education in my paper. This really is more or less a continuation of a paper which I read some years ago before the National Society, where I specified what I meant by preliminary education. It is not a question of quantity, but a question of quality, and that is precisely the fault I find with the forty-eight counts. I did not mean seventy-four subjects, but seventy-four branches. English counts for twelve, German three, French three, Latin four, science of all kinds eight, history eleven, other studies twelve, which make seventy-four branches, and not seventy-four subjects. That was a misunderstanding.

What would it avail dentists to know civics or economical history, commerce, home science, and such subjects. It is a satisfaction to have these, of course, as preliminary education, but they are irrelevant to our profession.

Dr. Truman said the reason why the older practitioners were made dentists after two years was not because they had more preliminary education, but they were selected by their preceptors. They were told, You are, or you are not, fitted to become a dentist.

Two or three years ago I had a student, an M.D., and it took him two years to determine the difference between a round and a square hole.

With respect to English, I am reminded of a story. An Hungarian said to an American, "Your English is very difficult. You have such long terms; for instance, you say 'inconvenience,' five long syllables." The American asked him how he would say it, and he answered, "Netlikaplongcheck."

Specific education ought to begin early in life. The child should be taught as early as possible, so that the teacher can train it and lead it to the profession where his natural abilities indicate. If the boy is unable to use his fingers in kindergarten work, he will probably not make a dentist; and if he has manual skill in the kindergarten, he may make an excellent surgeon or an excellent dentist, but may make a very poor preacher.

Dr. Kirk remarked that teachers are compensated too poorly to make ideal teachers. In the secondary schools the teachers are poorly paid, and frequently are of poor quality. The time will come when the American schools will be upon the same level with the European schools, but not until they have been separated from politics will there be an advance in this system of education.

I have been handed a question to answer: If four years are required for dentists, how much time should be given for medicine? That is a question I would not like to answer offhand. Some time ago a gentleman asked me, "If you were a member of a committee in Albany or Philadelphia, and some one offered you two million dollars to do nothing, what would you do?" I said, "I would sleep over it."

In conclusion, permit me to thank you for the invitation to be present and the good time I have had.

On motion, a vote of thanks was tendered by the Society to Dr. Hofheinz and to Dr. Litch for his discussion of the paper.

Otto E. Inglis,
*Editor Academy of Stomatology.*

